# Depression among TB patients and associated factors in Kathmandu Valley, Nepal

**DOI:** 10.1017/gmh.2019.28

**Published:** 2020-01-22

**Authors:** P. Shrestha, U. K. Subba, M. Brouwer, A. C. Sweetland

**Affiliations:** 1 Health Research and Social Development Forum (HERD), Thapathali, Kathmandu, Nepal; 2 Clinical Psychology, Department of Psychology and Philosophy, Tri Chandra Multiple Campus, Kathmandu, Nepal; 3 Department of Psychology and Philosophy, Tri Chandra Multiple Campus, Kathmandu, Nepal; 4 PHTB Consult, Public Health and Tuberculosis Control, Tilburg, Netherlands; 5 Department of Psychiatry, Columbia Vagelos College of Physicians and Surgeons/New York State Psychiatric Institute, New York, USA

**Keywords:** Depression, DOTS, Kathmandu, Patients, Tuberculosis

## Abstract

**Introduction:**

When tuberculosis (TB) and depression co-occur, there is greater risk for comorbidities, disability, suffering, and health-related costs. Depression is also associated with poor treatment adherence in patients with TB. The major aim of this study was to assess the symptoms of depression and associated factors among TB patients currently receiving directly observed treatment short-course (DOTS) treatment.

**Methods:**

A cross-sectional study was conducted among TB patients currently undergoing treatment in 27 DOTS centers in three districts of Kathmandu Valley. The study included 250 TB patients within 2 months of treatment initiation, aged 18 years and above. The previously validated Nepali Patient Health Questionnaire (PHQ-9) was used to screen for depression and semi-structured interviews were conducted to collect socio-demographic information and other factors related to TB and/or depression. Data analysis was conducted using IBM SPSS Statistics version 20.

**Results:**

The study found the mean PHQ Score to be 2.84 (s.d. 4.92, range 0–25). Among the respondents, 10% (*n* = 25) had PHQ-9 scores ⩾10, suggestive of probable depression. Multivariate linear regression indicated that depressive symptoms were significantly associated with being separated/widowed/divorced (*p* = 0.000) and having lower education (0.003). In addition, smoking (*p* = 0.02), alcohol use (*p* = 0.001), and experience of side effects from TB medications (*p* = 0.001) were risk factors for higher PHQ-9 scores.

**Conclusions:**

Our findings suggest that patients on TB treatment have higher risk of depression and efforts should be made by the National Tuberculosis Program to address this issue.

## Background

Depression may affect as many as half of all individuals with tuberculosis (TB) (Sweetland *et al*., 2014), which presents significant challenges to the management of these comorbid illnesses. Anxiety and depression are frequently co-occurring among patients with TB and are associated with poor adherence to anti-TB medications (Ige and Lasebikan, 2011; Duko *et al*. 2015) and higher mortality (Koyanagi *et al*., 2017). When TB and depression co-occur, there is a greater risk for other comorbidities, patients may suffer more, and associated cost increases (Basu *et al*., 2012). Depression may also pose an increased susceptibility to TB reactivation by compromising immunity or through neglected self-care (Sweetland *et al*., 2017). Thus, depression may be an unrecognized driver of the global TB and multidrug-resistant TB (MDR-TB) epidemics (Sweetland *et al*., 2014; Koyanagi *et al*., 2017).

A number of studies have assessed co-morbidity of depression and anxiety with TB (Amreen and Rizvi, 2016). A research study in Nigeria showed that 41.9% TB patients had depressive symptoms. Likewise, research conducted in India showed that the prevalence of depression among TB outpatients was 49%, and 54% in hospitalized TB patients (Adem *et al*., 2014). Similarly, evidence from cross-sectional studies in several African countries found the prevalence of comorbid depression among TB patients in a range from 10% to 52% (Ambaw *et al*., 2015). Two studies in Nepal have assessed the prevalence of depression among patients with drug-susceptible TB and MDR-TB, respectively. At an urban directly observed treatment short-course (DOTS) center in Kathmandu, of the 150 TB patients included in the sample, 18% had depression (Devkota *et al*., 2016). The frequency of mild depression was 16 (11%), for moderate depression was 6 (4%), for severe depression was 3 (2%) and that for very severe depression was 2 (1%). All cases of depression required medical attention. In a recent sample of 135 patients with MDR-TB in Nepal, the prevalence of depression and anxiety were 22.2% and 15.6%, respectively (Walker *et al*., 2019).

The co-occurrence of depression and TB can negatively impact quality of life, health care costs and self-care, as well as decreased immunity; all of these can affect patients' adherence to TB treatment and TB outcomes (Duko *et al*., 2015; Kunal *et al*., 2016; Sweetland *et al*., 2017; Ambaw *et al*., 2018). Poor treatment adherence not only increases the risk of negative TB outcomes, but also poses a public health risk given the potential for community transmission and/or development of drug resistance (Ambaw *et al*., 2017; Sweetland *et al*., 2017). Psychosocial concerns such as stigma, isolation, limited social support, helplessness, and other psychological reactions to the disclosing TB diagnosis, as well as medication side effects, all adversely impact treatment adherence (Mounika *et al*., 2016).

Given the limited availability of data on the prevalence of depression among TB patients in Nepal, this study was conducted with the aim of identifying the prevalence of depression among TB patients and the social factors associated with it.

## Methods

### Study design and population

A descriptive cross-sectional study was conducted among TB patients currently under treatment in DOTS centers in different treatment centers of Kathmandu Valley to study the prevalence of depression and associated factors. A total of 27 DOTS centers were selected from the three districts of Kathmandu Valley, including 10 from Kathmandu, 11 from Lalitpur, and 6 from Bhaktapur. Urban centers with higher caseloads were preferentially selected to achieve the desired sample size. Patients meeting the inclusion criteria (new adult patients within 2 months of initiating TB treatment) were recruited from these DOTS centers until the desired sample size was achieved. Medications and treatment protocols were identical across all DOTS sites.

### Sampling and inclusion criteria

Given that depression may be highest at baseline (Yen *et al*., 2015; Ambaw *et al*., 2017; Dasa *et al*., 2019) and may be higher in patients with MDR-TB (Vega *et al*., 2004), only new TB patients (never treated before) aged 18 years and above in the first 2 months of DOTS treatment (intensive phase) through the National TB Program were considered for the study. In order to maximize comparability across the sample, individuals being re-treated for TB (possible MDR-TB) or who had received more than 60 days of treatment (continuation phase) were excluded.

The sample size was calculated using the following formula (Naing *et al*., 2006):
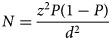
where *N* = sample size, *Z*^2^ = *Z* statistic for a level of confidence (95%), *P* = prevalence (18%) (Devkota *et al*., 2016), and *d* = precision (5%).

Based on the above mentioned values, the sample size (*N*) was calculated as follows:
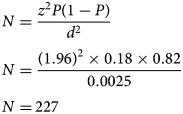


Adding 10% non-response rate,
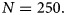


### Measurement

The previously validated Nepali Patient Health Questionnaire (PHQ-9) (Kohrt *et al*., 2016) was used to screen for depression (dependent variable). The PHQ-9 was originally developed to be a self-administered screening tool for patients in medical settings based on the PRIME-MD (Kroenke *et al*., 2001), but is often administered via interview in low literacy settings (Kohrt *et al*., 2016). Using a cutoff score of ⩾10, the Nepali PHQ-9 has demonstrated a sensitivity of 0.94 and a specificity of 0.80 (Kohrt *et al*., 2016). Independent variables included sociodemographic characteristics, social stigma, perceived social support, medication adherence, and TB-related side effects. To assess social stigma, patients were asked several questions based on prior studies including if they had disclosed their TB status to family and friends, had direct experiences of stigma or discrimination, felt guilty or shy about their TB status, and/or felt helplessness or loneliness due to their diagnosis. Patients were also asked to rate their perceived social support, whether they had ever forgotten to take their medications (adherence), and if they experienced any TB-related side effects.

### Data collection

The health workers of the respective DOTS centers were oriented about the study as well as informed about the use of PHQ-9. Trained research assistants administered the PHQ-9 to assess the frequency and severity of depressive symptoms, followed by a semi-structured interview to obtain socio-demographic characteristics, TB-related information, and other factors.

Ethical approval was obtained from the Department of Psychology and Philosophy, Tri Chandra Multiple Campus, Kathmandu to conduct the study. Verbal informed consent was obtained from the participants prior to the study. The consent form was designed and approved before inclusion of participants in the study. The participants were informed about the purpose of the study, about their voluntary participation in the study and their right to leave the study at any time. Confidentiality was maintained at all levels of the study. The data collected were also kept confidential and used by the researcher for the study purpose only.

### Data analysis

The collected data was entered into IBM SPSS Statistics version 20. Given the strong overlap in TB and depressive symptoms, and the fact that the screening scale was previously validated among a non-TB sample, we decided to analyze the PHQ-9 data in two ways including logistic (depression as binary using a cut off score of 10) and linear (depression symptoms as continuous) regressions. The age group and educational level were treated as ordinal variables, whereas other sociodemographic variables were treated categorically, including sex (male = 0, female = 1), marital status, and occupation. For marital status we used ‘married’ as the default comparison group and created two dummy variables for ‘never married’ or ‘separated/widowed/divorced.’ For occupation, we compared ‘wage labor’ and ‘student’ to everything else.

## Results

### Sociodemographic characteristics of respondents

The sociodemographic characteristics of the sample are described in Table 1. The mean age the respondents was 32.69 (range 18–84, s.d. 14.86). The largest age group was between 18–24 years with 40% of the total respondents. The sex distribution was 55% male and 45% female. Approximately half (53%) were married and 44% had never married. Nearly one third (31.6%) of the respondents were current students. One quarter (26.0%) had completed their higher secondary education, and 20.8% had achieved a bachelor's degree or higher.

**Table 1.** Sociodemographic characteristics of respondents (*n*=250)

	Frequency	Percent
Age of respondents		
18–24 years	101	40.4
25–34 years	58	23.2
35–44 years	44	17.6
45–54 years	24	9.6
55–64 years	7	2.8
65 years and above	16	6.4
Sex of respondents		
Male	138	55.2
Female	112	44.8
Marital status of respondents		
Never married	110	44.0
Married	132	52.8
Others (separated, divorced, widow/ widower)	8	3.2
Educational status of respondents		
Illiterate	19	7.6
Informal education	12	4.8
Primary level	31	12.4
Lower secondary level	37	14.8
Secondary level	34	13.6
Higher secondary level	65	26.0
Bachelors level	39	15.6
Masters level and above	13	5.2
Occupational status of respondents		
Housewife	36	14.4
Government service	7	2.8
Non-government service	21	8.4
Business	40	16
Daily wages labor	29	11.6
Agriculture	22	8.8
Student	79	31.6
Not working	16	6.4

### Factors associated with TB

The sample included new cases of TB in the first phase of treatment (see Table 2). Of the total respondents, almost 50% (*n* = 124) were bacteriologically confirmed new pulmonary TB cases, 44.8% had extrapulmonary TB, and 5.6% were diagnosed by clinical pulmonary assessment. A total of 206 respondents (82.4%) were aware of their human immunodeficiency virus (HIV) status; of these, 94.6% (*n* = 195) were HIV negative and 4.4% (*n* = 11) were HIV positive. Approximately 69% respondents had disclosed their TB status to their family members, friends, and relatives and among those, 87% perceived good social support. Although only 5.6% (*n* = 14) of respondents reported experiences of stigma or discrimination because of TB, 13.2% (*n* = 33) respondents felt guilty or shy, 10.4% (*n* = 26) felt loneliness, and 7.2% (*n* = 18) respondents felt helplessness due to TB. A total of 6.8% of respondents (*n* = 17) self-reported having forgotten to take their TB medications at least one time during treatment so far and about 30% respondents (*n* = 74) had experienced some TB-related side effects at least once.

**Table 2.** TB associated factors (*n*=250)

	Frequency	Percent
Types of TB		
New PBC (pulmonary bacteriologically confirmed)	124	49.6
New PCD (pulmonary clinically diagnosed)	14	5.6
New EP (extra pulmonary)	112	44.8
HIV infection		
Yes	11	4.4
No	195	78.0
Unknown	44	17.6
Known status of TB among family, friends and relatives		
Yes	172	68.8
No	78	31.2
Perceived support from family, friends and relatives		
Yes	150	87.2
No	22	12.8
Feeling of stigma or discrimination due to TB		
Yes	14	5.6
No	236	94.4
Feeling of guilty or shy due to TB		
Yes	33	13.2
No	217	86.8
Feeling of loneliness due to TB		
Yes	26	10.4
No	224	89.6
Feeling of helplessness due to TB		
Yes	18	7.2
No	232	92.8
Forgotten to take TB medications (at least one time during the course of treatment)		
Yes	17	6.8
No	233	93.2
Experience of side effects from TB medications (at least one time during the course of treatment)		
Yes	74	29.6
No	176	70.4

### PHQ-9 score and depression

Among the respondents, 10.0% (*n* = 25) had scores above the cut off of 10, suggestive of probable depression. Forty-four patients (17.6%) reported that depressive symptoms negatively impacted their daily lives in their work, self-care, care for and/or relationship with others. When asked whether they perceived that people with TB had greater risk for depression, 18.0% (*n* = 45) agreed. Most patients (97.2%) believed that their TB would be cured (see Table 3).

**Table 3.** PHQ score and depression (*n*=250)

	Frequency	Percent
Presence of symptoms of depression (based on PHQ score ⩾10)		
Yes	25	10.0
No	225	90.0
How often in the past two weeks have the problems affected your work, self-care and your care for family and your relationship with others?		
Never	206	82.4
Sometimes	29	11.6
Often	15	6
Patient's perception on TB patients at risk of depression		
Yes	45	18.0
No	205	82.0
Patient's perception on curability of their TB		
Yes	243	97.2
No	7	2.8
		

### Association between PHQ-9 score and socio-demographic and TB-related factors

Bivariate linear (continuous regression) analysis found that the higher PHQ score was associated with older age (*p* = 0.009), marital status [never married (*p* = 0.008) and separated/widowed/divorced (*p* = 0.000)], and occupation [student (*p* = 0.027)]. When controlling for other sociodemographic factors through multivariate linear (continuous regression) analysis, age and occupation were no longer significant, and only being separated/widowed/divorced and having lower education were significant risk factors for higher PHQ-9 scores (*p* = 0.000 and *p* = 0.003, respectively). When treating PHQ-9 score as binary variable indicating the presence or absence of probable depression (above and below 10), multivariate logistic (binary regression) analysis found the same pattern of results (Table 4). Additional risk factors for high depressive symptoms were current smoking (*p* = 0.02), current drinking (*p* = 0.001), and having experienced TB-related side effects (*p* = 0.001) (Table 5).

**Table 4.** Relationship of PHQ score and symptoms of depression with socio demographic variables

Constant	Bivariate linear (continuous) regression			Multivariate linear (continuous) regression			Multivariate logistic (binary) regression		
	B	s.e.	*p* Value	B	s.e.	*p* Value	B	s.e.	*p* Value
	–	–	–	4.817	2.955	0.104	1.674	0.186	0.000
Age group	0.555	0.21	0.009*	−0.026	0.278	0.927	0.016	0.017	0.362
Sex	0.428	0.626	0.495	0.015	0.627	0.982	0.014	0.039	0.726
Marital status									
Never married	−1.653	0.619	0.008*	−0.647	0.88	0.463	0.072	0.055	0.194
Separated/widowed/divorced	8.296	1.69	0.000*	6.880	1.729	0.000*	−0.357	0.109	0.001*
Education	−0.323	0.168	0.055	−0.568	0.192	0.003*	0.029	0.012	0.016*
Occupation									
Student	1.474	0.663	0.027*	−0.319	0.952	0.738	0.043	0.06	0.471
Daily wage laborers	−0.723	0.972	0.458	0.462	1.013	0.649	−0.019	0.064	0.761

* Statistically significant.

**Table 5.** Relationship of PHQ score with TB related factors

TB related factors	*N*	%	B	s.e.	*p* Value
Type of TB (*n* = 250)					
Extra pulmonary TB	112	44.8%	0.331	0.626	0.597
Pulmonary TB	138	55.2%			
HIV status (*n* = 206)					
Positive	11	5.3%	1.897	1.468	0.198
Negative	195	94.7%			
Experience of side effects from TB medications (*n* = 250)					
Yes	74	29.6%	2.18	0.668	0.001*
No	176	70.4%			
Forgotten to take TB medications (*n* = 250)					
Yes	17	6.8%	0.799	1.236	0.519
No	233	93.2%			
Disclosure of TB status to family and friends (*n* = 250)					
Yes	172	68.8%	−0.991	0.626	0.597
No	78	31.2%			
Current smoking status (*n* = 250)					
Yes	12	4.8%	1.379	0.591	0.02*
No	238	95.2%			
Current alcohol use status (*n* = 250)					
Yes	21	8.4%	1.733	0.516	0.001*
No	229	91.6%			

* Statistically significant.

## Discussion

The prevalence of depression in our study was lower than findings from two other studies conducted in Nepal. In the first study among an urban sample patient with TB or MDR-TB, 18% suffered from some levels of depression requiring medical attention (Devkota *et al*., 2016). In a more recent study among only MDR-TB patients, the prevalence of depression was estimated as 22.2%. It is unclear why the prevalence of depression was significantly lower in our sample (10%); one reason could be that we excluded MDR-TB patients, who have a greater risk for depression (Vega *et al*., 2004), or because we used a different depression screening tool.

In fact, estimating depression prevalence in TB patients using screening tools, in general, is inherently problematic. Given the strong overlap of symptoms between TB and depression, particularly physical symptoms (e.g. changes in appetite, sleep, or energy level), it is not clear if it is even valid to use the same cut-off score used in validation studies among non-TB patients (Castro Silva *et al*., 2018). Individuals with high scores on self-report measures of depressive symptoms and meet interviewer-rated psychiatric diagnostic criteria (true positives) differ in important ways from individuals who obtain similarly high scores on self-report measures of depressive symptoms but do not meet interviewer-rated criteria for a diagnosis of depression (false positives) (Gotlib *et al*., 1995). That said, the PHQ-9 is one of the most widely used globally to screen for depression among TB patients. Compared to other studies using the PHQ-9 among TB patients, the prevalence of depression in our sample was lower than samples from Cameroon (25%) India (42%), Ethiopia (54%), Pakistan (56%), and Brazil (62%) (Mandaknalli and Giriraj, 2015; Amreen and Rizvi, 2016; Kehbila *et al*., 2016; Ambaw *et al*., 2017; Castro Silva *et al*., 2018) but higher than one study in Nigeria (6.2%) (Issa *et al*., 2009). Other global studies using Hospital Depression & Anxiety Scale found similarly high prevalence estimates for depression including 47% (India), 46% (Pakistan), and 43% (Ethiopia) (Husain *et al*., 2008; Duko *et al*., 2015; Kunal *et al*., 2016). When examining depression and anxiety together, two studies found a prevalence of 72% (Pakistan) and 80% (India) (Mounika *et al*., 2016; Siddiqua and Aisha, 2016). In Nigerian study using the Hamilton Depression Rating Scale, the prevalence of moderate depression was 32% (Ige and Lasebikan, 2011).

In terms of sociodemographic correlates, we found that being separated/widowed/divorced and/or lower educational attainment were the only significant risk factors for depression in our sample. These findings differ from a cross-sectional, community-based dataset from 48 low and middle-income countries, in which the prevalence of depressive episodes among those with TB based on the Composite International Diagnostic Interview was 23.7%, and was significantly associated with older age, female sex, lower levels of wealth, smoking, and diabetes (Koyanagi *et al*., 2017). That said, low education and marital disruption are among the most common social correlates with depression across cultures based on the World Mental Health Survey (Kessler and Bromet, 2014).

TB-related stigma and discrimination are still highly prevalent worldwide, and are associated with greater risk for depression (Sweetland *et al*., 2017). In our sample, 31% patient did not disclose their TB status among family, and many endorsed experiencing feelings of guilt, loneliness, and helplessness due to TB. Several studies have explored TB-related stigma in Nepal. One study among 60 TB patients found that 63.3% (*n* = 38) of the subjects had experienced stigma (Aryal *et al*., 2012), while another study among 89 patients revealed 64% of people were stigmatized due to TB (Priyanka and Dahal, 2016). A qualitative study in Nepal that sought to identify the causes of TB-related stigma and self-discrimination found patient's fear of transmission of disease and fear of gossip as the two main contributors (Baral *et al*., 2007). Causes of discrimination by the community included fear of perceived risk of infection; perceived links between TB and poverty and low caste; perceived links between TB and disreputable behavior; and perceptions that TB was a divine punishment. Of note, some patients felt they were discriminated against by health workers (Baral *et al*., 2007).

## Conclusions

This study revealed that depression is a significant challenge for TB patients in Nepal, requiring attention by the NTP and TB providers. The WHO End TB strategy explicitly calls for integrated patient-centered care, including the treatment of mental disorders, in order to achieve TB elimination (Stop TB Partnership, 2016), and the WHO recently called for a TB and Mental Health collaborative framework to meet these needs (Sweetland *et al*., 2018). Furthermore, many NTP directors globally have expressed openness to integrating counseling and brief psychological interventions that have demonstrated effectiveness (Sweetland *et al*., 2019). Routine screening for depressive symptoms among TB patients, followed by a timely confirmatory diagnosis so such patients can receive treatment in order to improve the likelihood of positive TB outcomes (Sweetland *et al*., 2017). Operational research may guide programs how to best implement this across settings.
